# Recent advances in research on the force transmission mechanisms regulating endothelial function in the corpus cavernosum via fluid shear stress

**DOI:** 10.3389/fphys.2026.1949178

**Published:** 2026-10-26

**Authors:** Yuanyuan Wu, Jiayin Hu, Zhentao Lin, Runnan Xu, Zekai Chen, Luhao Xie, Min Chen, Zhiyue Wu, Qi Zhang, Chenxiang Lu, Bodong Lv

**Affiliations:** 1 The Second School of Clinical Medicine, Zhejiang Chinese Medical University, Hangzhou, Zhejiang, China; 2 Department of Urology, The Second Affiliated Hospital, School of Medicine, Zhejiang University, Hangzhou, Zhejiang, China

**Keywords:** endothelial cells, erectile dysfunction, hemodynamic forces, mechanotransduction, shear stress

## Abstract

Penile erection is a precisely regulated vascular physiological process in which cavernous endothelial cells (CCECs) play a pivotal role in mechanical sensing and signal transduction. As a central biomechanical cue, fluid shear stress (FSS) is transduced by diverse mechanoreceptors on CCECs—including the glycocalyx, primary cilia, Piezo1 ion channels, and the PECAM-1 mechanosensory complex—resulting in the activation of endothelial nitric oxide synthase (eNOS) and nitric oxide (NO) production. This narrative review synthesizes the unique hemodynamic microenvironment of cavernous sinuses and the mechanical dynamics during the erectile cycle, focusing on the molecular mechanisms of mechanosensors and downstream signaling pathways regulating eNOS activity. Furthermore, the article elucidates how pathological conditions, such as atherosclerosis, hypertension, diabetes, aging, and microvascular sclerosis, induce endothelial dysfunction and vascular erectile dysfunction (ED) through perturbed mechanical microenvironments or damage to sensory structures. Finally, this paper proposes that targeting mechanotransduction pathways may offer novel strategies for preventing and treating ED and delineates promising directions for future investigations.

## 1 Introduction

Penile erection is a reflexive process regulated by neurovascular mechanisms. It is also a highly coordinated hemodynamic event dependent on three pivotal mechanisms: arterial inflow augmentation via cavernous artery dilation; cavernous smooth muscle relaxation enabling sinusoidal expansion and blood accommodation; and venous outflow reduction through subalbugineal venous plexus compression (Virag et al., 2004; Abdelilah-Seyfried and Ola, 2024). Contemporary research predominantly revolves around the NO/cGMP biochemical cascade, with notable advancements achieved in this domain (Hurt et al., 2002). Notably, however, this paradigm—predominantly focused on biochemical cascades—often neglects the biomechanical milieu intrinsic to vascular wall architecture. Specifically, it overlooks the pivotal role of hemodynamic forces, particularly fluid shear stress (FSS), in maintaining cavernosal vascular homeostasis (Andersson and Wagner, 1995; Castela and Costa, 2016).

During penile erection initiation and maintenance, cavernous arterial and sinusoidal blood flow velocities undergo substantial fluctuations, inducing pronounced FSS (Virag et al., 2004; Abdelilah-Seyfried and Ola, 2024). Upon sensing shear stress, CCECs activate eNOS via cascading signaling pathways, culminating in NO production (Hurt et al., 2002). NO diffuses to neighboring cavernous smooth muscle cells, stimulating soluble guanylate cyclase (sGC) and elevating cyclic guanosine monophosphate (cGMP) levels. This, in turn, triggers protein kinase G (PKG) activation, ultimately mediating smooth muscle relaxation (Castela and Costa, 2016). Concurrently, arterial vasodilation and cavernous smooth muscle relaxation enhance blood inflow, while cavernosal engorgement mechanically restricts venous outflow, collectively culminating in erection attainment (Andersson and Wagner, 1995; Hurt et al., 2002). Thus, CCECs function as bidirectional regulators: biomechanical sensors of hemodynamic forces and vasomotor effectors modulating vascular tone through NO release (Cheng et al., 2023). This mechanotransduction process—converting mechanical stimuli into biochemical signaling—is pivotal for maintaining physiological erectile function.

Despite advancements in elucidating the biochemical regulatory mechanisms of the NO/cGMP pathway, the mechanosensory mechanisms underlying physical forces acting as upstream triggers in the corpus cavernosum remain inadequately characterized. Concurrently, the regulatory roles of biomechanical factors—such as FSS—in cardiovascular and cerebrovascular homeostasis have gained widespread recognition. Notably, studies have demonstrated that FSS directly modulates endothelial function via mechanosensitive channels (e.g., Piezo1 and integrins) on the cell membrane (Mehta et al., 2020; Deng et al., 2026). However, the unique compliance and sinusoidal architecture of the corpus cavernosum pose distinct challenges to deciphering its specialized sensing and transduction mechanisms. To date, direct investigations into FSS effects within the corpus cavernosum remain sparse. Nevertheless, pathological conditions like diabetes or hypertension often induce local low shear stress or impair mechanosensing, disrupting the mechanical microenvironment. This imbalance likely triggers endothelial dysfunction, culminating in vascular-induced ED (Wessells et al., 2006; Castela and Costa, 2016; Dela Justina et al., 2023). The paucity of direct research in this area has impeded the development of targeted therapies for vascular ED. Therefore, this study aims to bridge this gap by integrating vascular biomechanics principles. Through a narrative synthesis of canonical mechanosensors and eNOS-regulating pathways, we will infer mechanical alterations in ED-associated pathologies. Our goal is to establish a theoretical foundation for mechanotransduction research in the corpus cavernosum and inform translational strategies targeting the mechanical sensing axis for clinical innovation.

## 2 Hemodynamic environment of the corpus cavernosum

The corpus cavernosum represents a highly specialized microcirculatory system, in which the fulfillment of physiological functions is underpinned by both biochemical regulatory mechanisms and its unique biomechanical microenvironment, particularly the dynamic fluctuations in FSS that govern vascular homeostasis (Davies, 1995; Virag et al., 2004).

### 2.1 Basic properties of shear stress

In hemodynamics, shear stress (τ) refers to the tangential force exerted by blood flow friction on the endothelial surface of blood vessels. In the cavernous microvasculature, its magnitude is primarily determined by blood flow velocity and vessel caliber, and can be quantified using the wall shear stress relation derived from the Hagen-Poiseuille solution (τ = 4ηQ/πr³, where η represents blood viscosity, Q signifies volumetric flow rate, and r denotes vessel radius) (Mutlu et al., 2023). Note that this expression is the wall shear stress relation derived from the Hagen-Poiseuille solution; the Hagen-Poiseuille equation itself relates pressure drop to flow rate (ΔP = 8ηLQ/πr^4^). Shear stress is classified into physiological and pathological types based on hemodynamic conditions (Abaci et al., 2014). Physiological shear stress, varies by vessel type: in straight large arteries the time-averaged wall shear stress is 20–40 dyn/cm² (rising to ~40–50 dyn/cm² at peak systole), whereas venous shear stress is only 1–2 dyn/cm² (Davies, 1995). Laminar shear stress (LSS), i.e., unidirectional steady flow in straight vascular segments, plays a pivotal role in endothelial protection, vascular homeostasis maintenance, and the preservation of erectile function (Tzima et al., 2005; Choublier et al., 2022). These magnitudes derive from systemic arterial measurements; direct measurements of FSS within the cavernosal sinusoids have not been reported, and the arterial values applied to the corpus cavernosum in this review are therefore extrapolations. The closest human evidence is shear-stress-mediated vasodilation of the cavernous arteries (Virag et al., 2004). Conversely, pathological shear stress occurs in pathological contexts such as hemodynamic disturbances or vascular remodeling, presenting as low shear stress or oscillatory shear stress (OSS) (Yamawaki et al., 2005; Peiffer et al., 2013; Timmins et al., 2017; Kumar et al., 2018; Siasos et al., 2018). As a critical biomechanical cue, shear stress modulates physiological and pathological processes through mechanoreceptor activation, signal transduction initiation, and downstream biological effects encompassing inflammation, oxidative stress, and NO production (Yamawaki et al., 2005; Chao et al., 2018). Given its profound impact on cavernous endothelial function, shear stress has emerged as a central determinant in the pathogenesis of ED (Azadzoi et al., 2005).

### 2.2 Fluid dynamics in the cavernous sinuses

Unlike the relatively stable laminar flow environment in large vessels, the corpus cavernosum of the penis is characterized by a reticular topological architecture formed by irregular, interconnected sinusoidal lacunae. This intricate geometric configuration gives rise to profound spatiotemporal heterogeneity in shear stress profiles (Chiu and Chien, 2011; Gutwein et al., 2025). As blood jets from high-resistance helicine arteries into low-resistance, expansive sinusoidal spaces, the abrupt dilation of the flow channel, coupled with the geometric tortuosity of sinusoidal walls, induces complex local flow perturbations, including regions of decelerated flow, flow separation, and transient vortical formations (Chiu and Chien, 2011; Fung, 2013). Moreover, during most of the erectile cycle the sinusoids constitute a low-flow, quasi-venous compartment, so arterial shear-stress magnitudes cannot be assumed to prevail on sinusoidal endothelium. These hemodynamic features indicate that CCECs are not subjected to unidirectional laminar loading, but instead experience a dynamic, heterogeneous fluid mechanical milieu. This biomechanical complexity necessitates CCECs to possess exquisite mechanosensitivity to discriminate physiological laminar stimuli from pathological turbulent disturbances (Chien et al., 1998). Experimental evidence confirms that physiological-magnitude shear stress elicits pro-homeostatic responses in CCECs, including anti-apoptotic and vasodilatory phenotypes. Conversely, hemodynamic impairments (e.g., reduced flow due to atherosclerosis) induce endothelial oxidative stress under conditions of low shear stress (Chien, 2008; Chao et al., 2018). Collectively, these anatomically determined hydrodynamic features define the physical constraints governing cavernous microcirculatory homeostasis and provide a spatial framework for understanding endothelial dysfunction in pathological contexts (Rosato et al., 2013).

### 2.3 Mechanical changes during the erectile cycle

The FSS within the corpus cavernosum does not remain static but undergoes characteristic “step-like” dynamic fluctuations across the distinct physiological phases of the erectile cycle. This biomechanical modulation plays a pivotal role in maintaining the functional homeostasis of cavernous sinusoidal endothelial cells (Virag et al., 2004; Wessells et al., 2006; Chien, 2008) (**Figure 1**). During the initiation phase, neural signaling triggers rapid arterial dilation, precipitating a surge in blood flow velocity within milliseconds (Andersson and Wagner, 1995). As predicted by Poiseuille’s law, this abrupt hemodynamic shift subjects CCECs to an intense transient shear stress pulse (Peak FSS). The absolute amplitude of this peak within the sinusoids has not been measured directly and is extrapolated from arterial hemodynamics. This mechanical impulse acts as a central trigger for mechano-chemical transduction, rapidly inducing conformational shifts in endothelial mechanoreceptors (e.g., glycocalyx and Piezo1), which in turn activate eNOS phosphorylation and promote NO release (Andersson and Wagner, 1995; Abaci et al., 2014). As the erection transitions to the maintenance and rigidity phases, compression of the subalbugineal venous plexus elevates intracavernous pressure, markedly attenuating blood flow velocity and causing FSS to plummet from its peak value to a stabilized low plateau (Wessells et al., 2006). This biomechanical transition is characterized by a modal shift—from tangential shear stress dominance to vertical hydrostatic pressure loading—signaling a functional switch in CCECs from active signal transduction to barrier maintenance (Wang et al., 2020b). These rhythmic FSS oscillations confer physiological resilience: stable laminar flow sustains high eNOS expression while suppressing pro-inflammatory cytokine synthesis in endothelial cells (Chien, 2008). Clinically, patients with vascular ED secondary to diabetes or atherosclerosis exhibit a defining pathomechanical feature: the “attenuation of mechanical signal fluctuation amplitude” (Wessells et al., 2006; Cheng et al., 2023). Low or disturbed shear stress is established as a driver of impaired endothelial function, reduced NO bioavailability, and inflammation (Yamawaki et al., 2005; Chao et al., 2018); however, it has not been directly demonstrated that attenuation of the proposed FSS peaks during the erectile cycle desensitizes or degrades Piezo1 or other mechanosensors in CCECs. We therefore hypothesize that chronic exposure to persistently low shear stress, or absence of effective peak pulses, desensitizes or degrades endothelial mechanosensors, potentially driving CCECs toward a pathological phenotype characterized by impaired NO bioavailability and inflammation, and that this biomechanical dysregulation may underpin the progression of vascular-origin ED (Hoey et al., 2012; Dela Justina et al., 2023).

**Figure 1:**
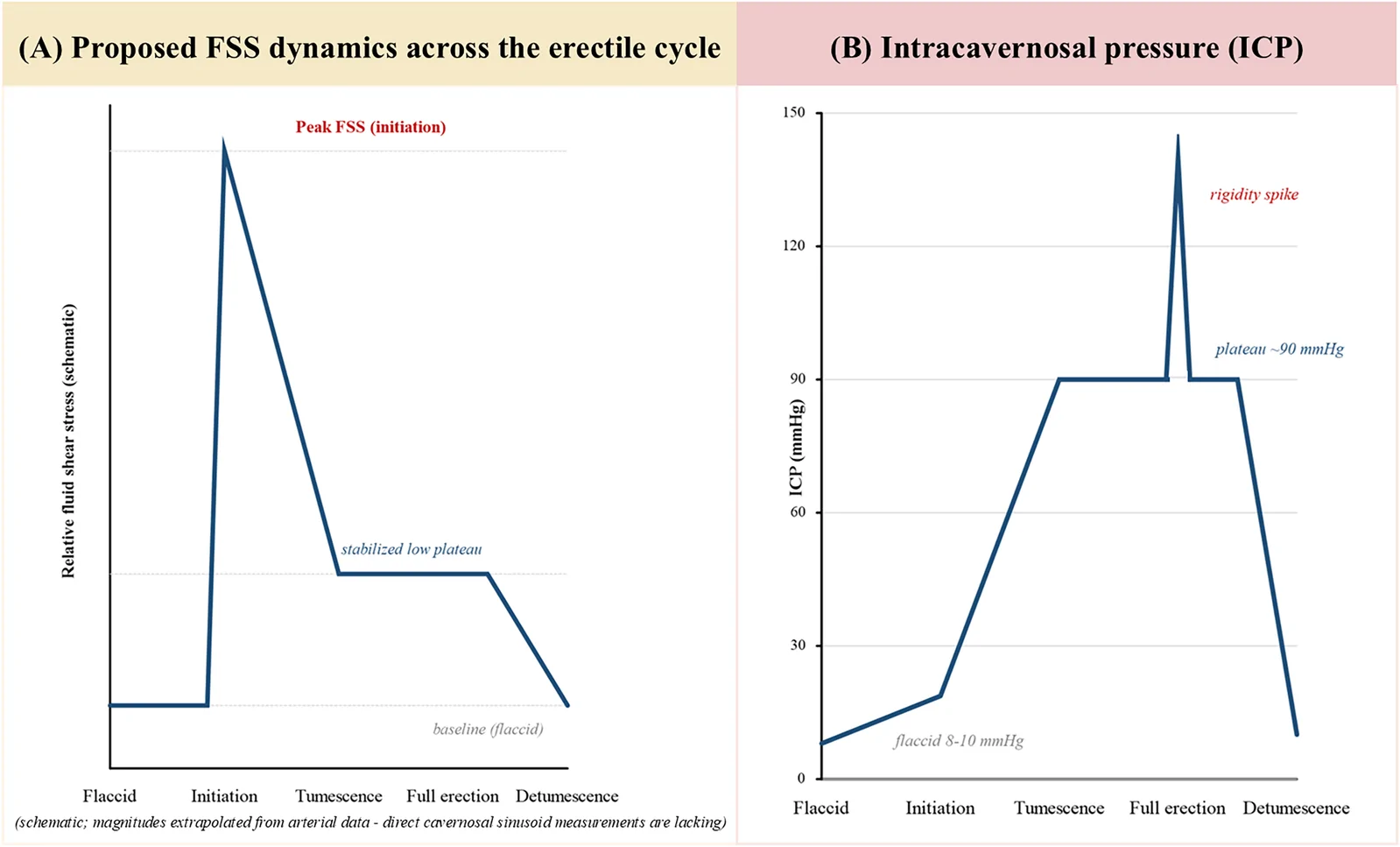
Hemodynamics of erectile cycle and cavernosal sinusoids. **(A)** Proposed FSS dynamics across the erectile cycle (flaccid, initiation peak, tumescence, full erection plateau, detumescence). **(B)** ICP (mmHg). In **(A)**, the proposed FSS peak and its subsequent decline are inferred from known hemodynamic changes during erection, rather than from direct measurements of sinusoidal FSS; panel **(A)** is therefore intended as a conceptual schematic rather than an established physiological pattern. Some illustrations in this picture were created by BioRender (BioRender.com).

## 3 The molecular basis of mechanical sensors in cavernous endothelial cells

Before detailing individual sensors, it is important to state what has—and has not—been demonstrated directly in the corpus cavernosum. Shear-stress-induced NO production has been demonstrated in cultured human CCECs (Wessells et al., 2006), shear-mediated vasodilation in human cavernous arteries (Virag et al., 2004), and functional Piezo1 expression in rat cavernosal tissue and the pudendal artery (Dela Justina et al., 2023); glycocalyx degradation is well documented in diabetic and aging vasculature (Vlahu et al., 2012; Liu et al., 2020; Wang et al., 2020b; Wang et al., 2020a), with functional cavernosal consequences (Wessells et al., 2006). By contrast, the roles of the PECAM-1/VE-cadherin/VEGFR2 complex, TRPV4 and primary cilia have been established in other endothelial types, and their operation in CCECs—although mechanistically plausible—remains to be demonstrated (see **Table 1**).

**Table 1:** Summary of core mechanosensors and their pathophysiological features in cavernosal endothelial cells.

Mechanosensor	Subcellular localization	Activation mechanism & physical stimuli	Signaling pathways	Pathological alterations	Ref	Direct evidence in CCECs
Piezo1	Plasma membrane	Membrane stretch/Local fluid shear stress (FSS)	Ca^2+^ influx→Akt→eNOS	Aging/fibrosis (high pre-stress) causes an elevated activation threshold (mechanosensory desensitization); hypertension leads to overactivation and eNOS uncoupling.	(Coste et al., 2010; Wu et al., 2017; Beech and Kalli, 2019; Douguet et al., 2019; Swain and Liddle, 2021; Wang et al., 2021; Yao et al., 2022; Dela Justina et al., 2023; Lai et al., 2023)	Yes
Endothelial Glycocalyx	Apical membrane	Direct exposure to luminal fluid shear and friction	Mechanical force transmission→Cytoskeleton & membrane receptor clusters	Diabetes (AGEs) and atherosclerosis (disturbed flow) induce severe degradation and physical shedding of core components.	(Pahakis et al., 2007; Reitsma et al., 2007; Weinbaum et al., 2007; Vlahu et al., 2012; Liu et al., 2020; Wang et al., 2020b; Wang et al., 2020a; Bartosch et al., 2021; Sun et al., 2023)	Partly
Primary Cilia	Apical membrane	Responds to flow-induced bending forces	Polycystin-1/2→Intracellular calcium release	Aging, chronic high stress, or oxidative stress causes shortening and numerical reduction, impairing weak shear stress detection.	(Nauli et al., 2003; Singla and Reiter, 2006; Hoey et al., 2012; Kallakuri et al., 2015; Luu et al., 2018; Saternos et al., 2020)	No
PECAM-1/VEGFR2	Intercellular adherens junctions	Intercellular mechanical tension and deformation induced by FSS	Tyrosine phosphorylation→PI3K/Akt→eNOS activation	High oxidative stress in diabetes disrupts complex stability, resulting in impaired signal transduction and diminished NO production.	(Bivalacqua et al., 2003; Fleming et al., 2005; Tzima et al., 2005; Coon et al., 2015)	No
Integrins	Basal membrane	Increased ECM stiffness and physical traction	FAK activation→RhoA/ROCK→eNOS inhibition	Fibrosis/aging stiffens the matrix, abnormally activating integrins to generate pathological intracellular “pre-stress” and exacerbate fibrosis.	(Urbich et al., 2000; Hynes, 2002; Campbell and Humphries, 2011; Du et al., 2016; Liu et al., 2018; Yu et al., 2020; Lin et al., 2023; Katoh, 2025; Liu et al., 2025)	No

Direct evidence in CCECs: Yes, demonstrated in cavernosal tissue/ECs; Partly, cavernosal functional evidence with mechanism inferred from other EC types; No, extrapolated hypothesis.

### 3.1 Membrane-bound mechanoreceptors: the glycocalyx and primary cilia

The endothelial glycocalyx (EG) is a carbohydrate-rich, mesh-like structure covering the apical membrane surface of sinusoidal endothelial cells, primarily composed of proteoglycans (such as syndecans) and glycosaminoglycans (such as hyaluronic acid and heparan sulfate) (Reitsma et al., 2007; Weinbaum et al., 2007). Heparan sulfate accounts for 50%-90% of the glycosaminoglycans in the glycocalyx and is involved in mediating various functions of endothelial cells, including the force transmission process of shear stress (Bartosch et al., 2021). As the first “physical barrier” in contact with shear stress, the EG performs a critical role in mechanical signal transduction. When blood flows through the sinusoids, the EG captures minute fluctuations in shear stress through the physical deformation of its long-chain structure and transmits this mechanical energy to the cytoskeleton and transmembrane proteins (such as PECAM-1), thereby triggering the activation of downstream eNOS (Pahakis et al., 2007; Weinbaum et al., 2007). In vascular-origin ED caused by diabetes or hyperlipidemia, the degradation of EG often serves as an early marker of endothelial dysfunction—once this mechanosensory layer is compromised, endothelial cells are unable to perceive effective shear stress signals even under normal hemodynamic conditions, leading to a significant reduction in NO release (Vlahu et al., 2012; Wang et al., 2020b; Wang et al., 2020a). Unlike the widespread distribution of EG, primary cilia are single tubular organelles projecting from the surface of CCECs and are considered mechanosensors that are highly sensitive to low-intensity shear stress (Singla and Reiter, 2006; Hoey et al., 2012). In the microenvironment of the cavernous sinuses, where flow velocity is highly variable, primary cilia sense fluid displacement through their internal microtubule structures and, in concert with the polycystin-1/2 complex, regulate intracellular Ca^2+^ homeostasis (Nauli et al., 2003; Saternos et al., 2020). Although research on primary cilia in CCECs is still in its early stages, evidence suggests that cilia loss or dysfunction is closely associated with pathological remodeling of micro vessels, which may provide a new perspective on how flow abnormalities within the cavernous sinuses lead to endothelial damage (Kallakuri et al., 2015; Luu et al., 2018).

### 3.2 Force-sensitive ion channels: the Ca²^2^ channel family centered on Piezo1 (including TRPV4)

Piezo1 is a non-selective cation channel with a unique “three-bladed paddle” structure. Owing to its mechanical sensitivity, it responds to fluid shear stress, and functional Piezo1 has been demonstrated in the corpus cavernosum and pudendal artery (Dela Justina et al., 2023), thereby enabling “force-electric/force-chemical” coupling (Coste et al., 2010; Douguet et al., 2019). During the engorgement phase of erection, when transient blood flow impacts the cavernous sinus wall, FSS-induced changes in cell membrane tension cause a conformational flip in the Piezo1 paddle structure, causing the channel to open instantaneously and mediate a massive influx of extracellular Ca^2+^ (Dela Justina et al., 2023). This transient surge in Ca²^2^ signaling has a dual effect: on the one hand, it directly binds to and activates eNOS via the Ca²^2^/calmodulin (CaM) complex, leading to rapid NO release (Wu et al., 2017; Beech and Kalli, 2019); In systemic arteries, endothelial Piezo1 is required for flow-induced NO formation and vasodilation (Wang et al., 2016). On the other hand, Piezo1-mediated signaling can also establish lateral communication with the underlying cytoskeleton and focal adhesions, enhancing the structural stability of endothelial cells (Yao et al., 2022). In addition to Piezo1, transient receptor potential vanilloid 4 (TRPV4) is a force-sensitive channel activated by low-intensity shear stress that contributes to blood flow regulation and shear-induced vasodilation—as demonstrated in human umbilical vein endothelial cells (HUVECs) and rat carotid endothelial cells (Köhler et al., 2006; Swain and Liddle, 2021). Whether TRPV4 fulfils an equivalent role in cavernous sinus endothelial cells has not been established and remains an untested hypothesis. During phases of dramatic blood flow fluctuations, such as the engorgement phase of erection, we hypothesize that Piezo1 and TRPV4 transition from a parallel to a cascade mode: Piezo1, which responds first to mechanical signals, enhances TRPV4’s responsiveness through biochemical coupling, enabling efficient regulation of complex mechanical environments. However, if this cascade reaction—demonstrated in cultured endothelial cells—leads to Ca²^2^ overload due to excessive triggering, it can induce pathological damage such as endothelial dysfunction and cytoskeletal disruption (Swain and Liddle, 2021). From a pathophysiological perspective, downregulation of Piezo1 function or an increased threshold for mechanical signals (i.e., “desensitization”) is a key mechanism leading to the inability of cavernous endothelial cells to efficiently convert physical displacement into biochemical signals, thereby triggering vascular-derived ED. These two apparently contradictory scenarios—excessive Piezo1 activation versus downregulation/desensitization—are best reconciled by disease context: sustained overactivation predominates in hypertension, where Piezo1 is upregulated and chronically stimulated, whereas reduced Piezo1 function and an elevated activation threshold predominate in aging, fibrosis and chronic low-shear states. Direct evidence in the corpus cavernosum is available for agonist-induced Piezo1 activation (Dela Justina et al., 2023); the stage-dependent switching between these two modes remains to be verified experimentally in CCECs.

### 3.3 Adhesion protein complex: PECAM-1/VE-cadherin is the central axis of force transmission

At the junctions between adjacent CCECs, there exists a key mechanosensory complex composed of PECAM-1 (platelet-endothelial cell adhesion molecule-1), VE-cadherin (vascular endothelial cadherin), and VEGFR2 (vascular endothelial growth factor receptor 2) (Tzima et al., 2005). The architecture and function of this complex were established in cultured endothelial cells (Tzima et al., 2005; Coon et al., 2015); its operation in CCECs is inferred and, although mechanistically plausible, remains to be demonstrated directly. During biomechanical transduction, changes in cell membrane tension or cytoskeletal stress induced by shear forces are progressively transmitted along the cell surface toward the periphery, thereby concentrating the physical load on the junctional proteins at the interface. This alteration in stress distribution drives the reorganization of intermolecular forces within the complex, providing the kinetic basis for the transmembrane transduction of mechanical signals. Taking PECAM-1 as an example, when shear stress acts on the endothelial surface, PECAM-1, acting as a direct force transducer, undergoes a conformational change. Utilizing the scaffolding function of VE-cadherin, it rapidly transmits the mechanical signal to VEGFR2 and induces its autophosphorylation without the involvement of ligands, subsequently recruiting and activating the protein kinase Akt via phosphoinositide 3-kinase (PI3K), which ultimately triggers the phosphorylation and biochemical activation of eNOS, a pathway proposed, by extrapolation from cultured endothelial cells, to maintain vascular homeostasis in the corpus cavernosum during the non-erect state (Fleming et al., 2005; Coon et al., 2015). This “force-signal coupling” process constitutes the core cascade mechanism by which endothelial cells in the corpus cavernosum convert fluid shear stress from blood flow into NO-mediated vasodilatory signals (Bai et al., 2019). Furthermore, in pathological conditions characterized by chronic impairment of intracavernosal pressure, the dissociation or downregulation of this connecting complex leads to impaired endothelial barrier function and a pathological elevation in the mechanical perception threshold, thereby exacerbating the clinical manifestations of vascular-induced ED (Bivalacqua et al., 2003) (**Figure 2**).

**Figure 2:**
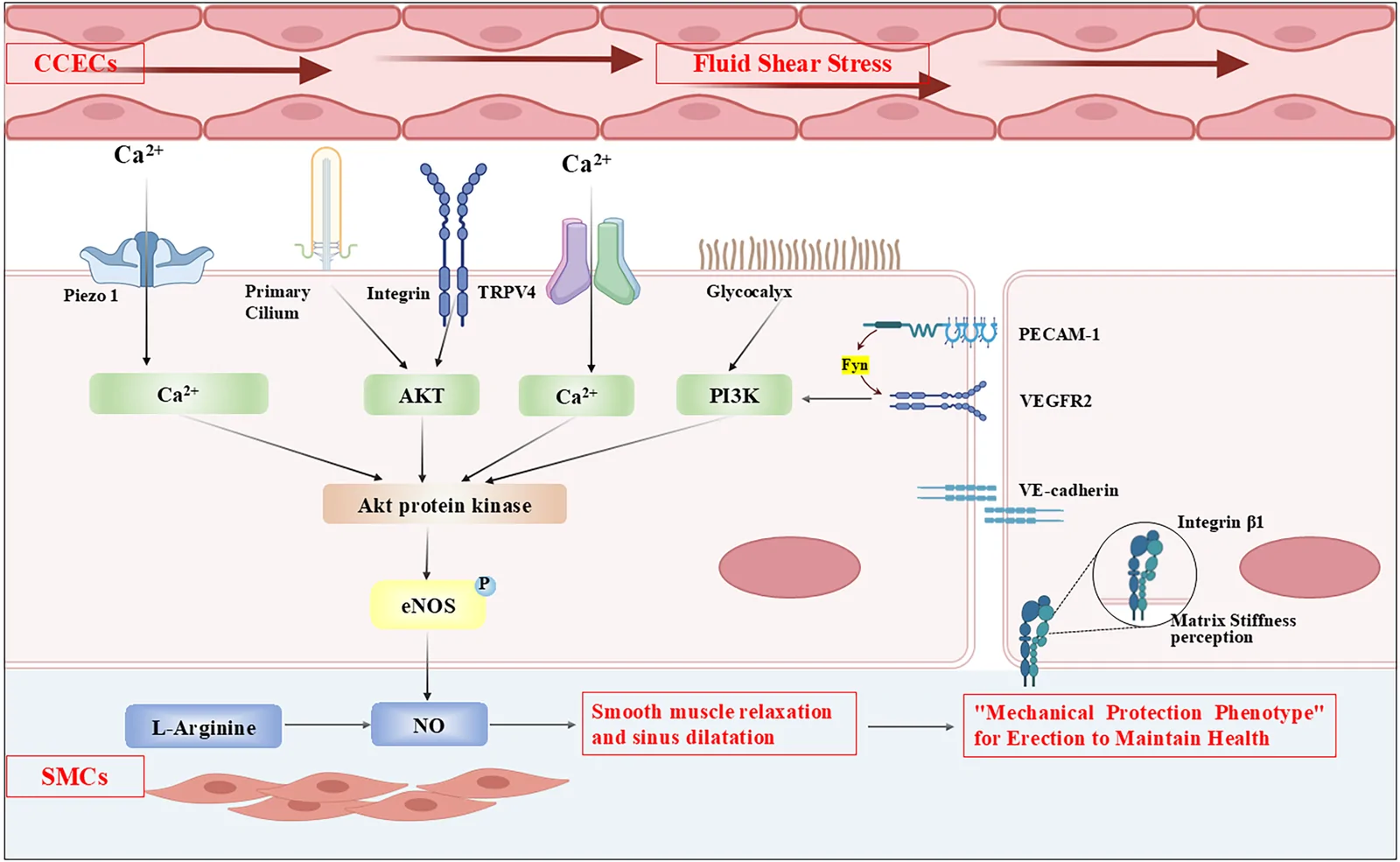
Transmission mechanism diagram of cavernous endothelial cells under fluid shear stress. Some illustrations in this picture were created by BioRender (BioRender.com).

### 3.4 Adhesion structures and the cytoskeleton: synergistic sensing by integrins and the cytoskeleton

Integrins, as the key transmembrane proteins mediating the adhesion of CCECs to the extracellular matrix (ECM), consist of two non-covalently bound subunits, α and β. Their head regions bind to the ECM, while their tail regions connect to intracellular F-actin, mediating cell-cell and cell-ECM recognition, adhesion, and communication (Hynes, 2002; Takagi and Springer, 2002). Not only can integrins sense shear stress transmitted from the cell membrane surface, but they can also detect changes in extracellular matrix stiffness (Urbich et al., 2000; Du et al., 2016). These findings derive from non-cavernosal endothelial and vascular models and are extrapolated to CCECs. Under mechanical stimulation, integrins densely clustered on the cell surface transmit mechanical signals to the cytoskeleton via adhesion complexes, promoting the polymerization of intracellular actin, enhancing cell adhesion and contraction, and subsequently activating downstream transcription factors to regulate cellular behavior (Katoh, 2025). Downstream of integrin engagement, the Hippo pathway effectors YAP/TAZ act as central mediators of stiffness- and flow-dependent endothelial phenotypes: laminar flow suppresses YAP/TAZ activity, whereas disturbed flow and matrix stiffening activate it to drive pro-inflammatory and pro-fibrotic gene programs (Wang et al., 2016; Wang et al., 2016). Whether YAP/TAZ function similarly in CCECs is untested. During the development of vascular-origin ED, the corpus cavernosum, influenced by factors such as chronic hypoxia, oxidative stress, or hemodynamic abnormalities, initiates pathological repair processes, manifested as smooth muscle cell phenotypic transformation and the release of pro-fibrotic factors (Zhang et al., 2018). These changes disrupt the dynamic balance between ECM synthesis and degradation, leading to a reduction in elastic fibers and excessive deposition of collagen, ultimately inducing corpus cavernosum fibrosis (Qabazard et al., 2021). As a key pathological feature of vascular-origin ED, fibrosis not only reduces corpus cavernosum compliance but also alters the physical microenvironment of endothelial cells (Leungwattanakij et al., 2003; Cui et al., 2018). During fibrosis, ECM stiffness increases due to abnormal collagen deposition, and integrins can detect abnormal mechanical signals transmitted from the basement membrane (Liu et al., 2025). This pathological mechanical feedback further induces cytoskeletal reorganization, activates contraction-related signaling pathways such as RhoA/ROCK, and inhibits eNOS activity, thereby accelerating the progression of fibrosis (Liu et al., 2018; Yu et al., 2020; Lin et al., 2023). Therefore, the synergistic interaction between integrins and the cytoskeleton not only serves as the structural basis for mechanical signal transduction but also constitutes the key molecular mechanism by which CCECs distinguish between “physiological fluid shear stress” and “pathological matrix stiffening” (**Table 1**).

## 4 Mechanical changes in biological systems under pathological conditions

As mentioned earlier, CCECs exhibit excellent “force-chemistry coupling” responsiveness. However, during the progression of vascular-origin ED, chronic pathological conditions not only damage blood vessels through biochemical pathways but also reshape the biomechanical environment of the corpus cavernosum at the biophysical level. The following sections will provide a detailed discussion of the biomechanical changes associated with different clinical pathological states (**Figure 3**). It is equally important to distinguish cause from consequence: in diabetes, hypertension, aging and atherosclerosis, impaired mechanotransduction may contribute to ED directly, or may arise secondarily once cavernosal blood flow declines; most plausibly, the two processes reinforce each other in a vicious cycle (reduced FSS → endothelial dysfunction → reduced NO → further impairment of blood flow) (Malek et al., 1999; Wessells et al., 2006; Chien, 2008; Chiu and Chien, 2011; Chao et al., 2018). Population-level causal-inference tools such as Mendelian randomization and co-localization analysis—recently used to demonstrate the causal vascular toxicity of the anti-inflammatory herbal medicine Indigo naturalis (Liu et al., 2025)—offer a complementary route to disentangling cause from consequence in vascular ED. Furthermore, endothelial cells propagate dysfunction to neighbouring cells through paracrine signals and extracellular vesicles (EVs) carrying inflammatory, pro-oxidant and pro-fibrotic cargo; EV-mediated spread of endothelial dysfunction is increasingly recognized in cardiovascular diseases and may likewise operate within the cavernous sinusoids (Jimenez-Trinidad et al., 2025).

**Figure 3:**
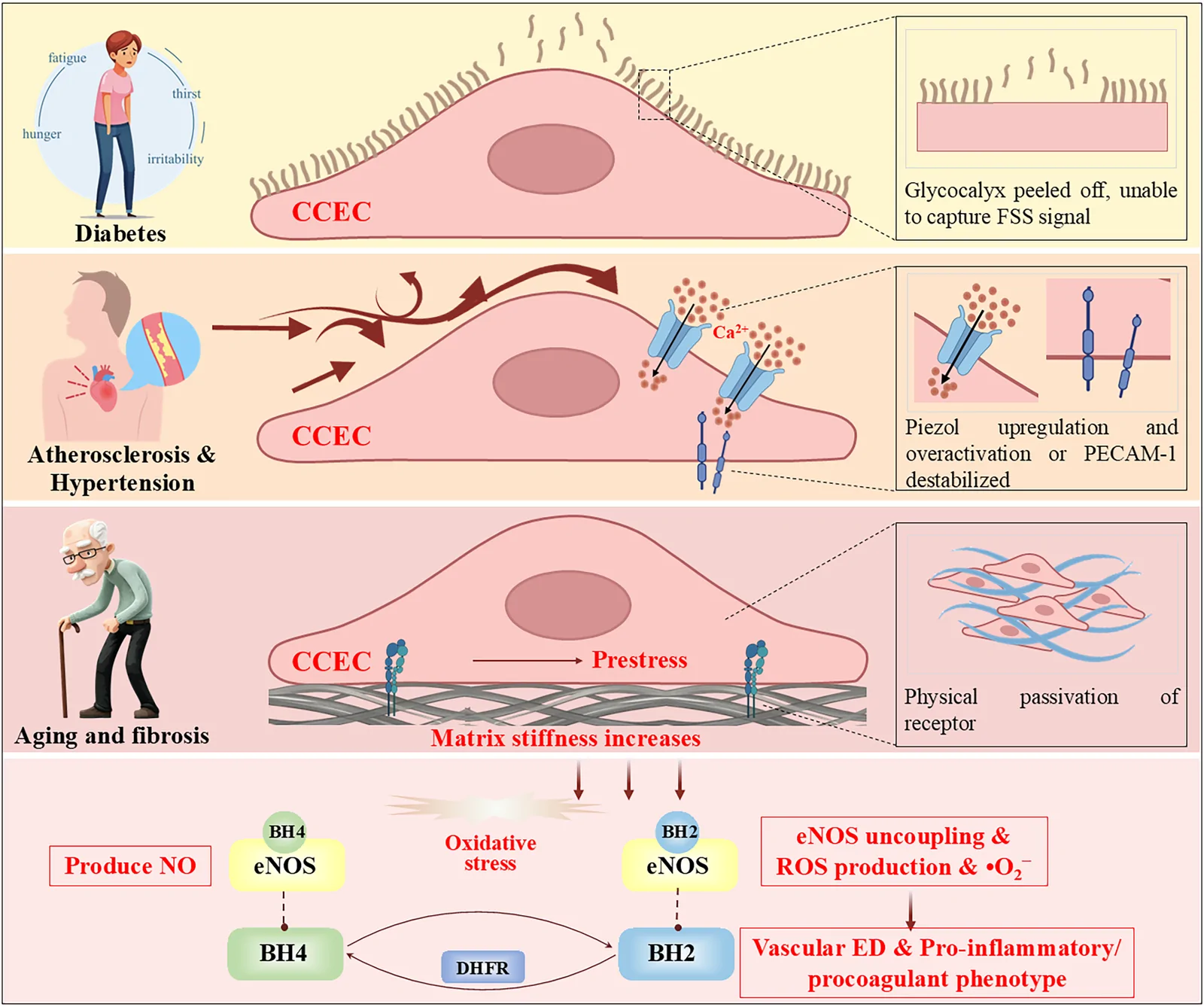
Mechanical perception dysfunction diagram in pathological state. Some illustrations in this picture were created by BioRender (BioRender.com).

### 4.1 Atherosclerosis: mechanical polarization from laminar to turbulent flow

Atherosclerosis induces a shift in hemodynamic forces from physiological “steady-state laminar flow” to pathological “local turbulent flow” by altering the luminal morphology of the cavernous spiral arteries (Chiu and Chien, 2011; Cheng et al., 2023). Under normal physiological conditions, high shear stress at the arterial level (>15 dyn/cm²) maintains endothelial cells in a quiescent, protective phenotype characterized by anti-proliferative, antioxidant, and anti-thrombotic properties (Davies, 1995; Niu et al., 2019). This atheroprotective threshold was established in systemic arteries (Malek et al., 1999); whether it applies within the cavernosal sinusoids remains unverified. However, plaques not only lead to insufficient perfusion of the distal cavernous sinuses but also cause significant flow separation at vascular bifurcations or distal to stenoses, creating regions of low FSS (typically <4 dyn/cm²) and OSS (Zarins et al., 1983; Malek et al., 1999). At the transcriptional level, laminar flow induces the atheroprotective transcription factors KLF2/KLF4, which suppress NF-kB-driven inflammatory programs and induce eNOS expression (SenBanerjee et al., 2004; Parmar et al., 2006); disturbed and low shear flow downregulates KLF2/KLF4, releasing the brake on pro-inflammatory polarization. These findings are established in systemic and coronary endothelium and are presumed, but unproven, to apply to cavernous endothelial cells. Studies in arterial endothelial cells show that long-term exposure to low FSS induces significant pro-inflammatory polarization; this phenotype is presumed to occur in CCECs as well: core components of the endothelial surface EG, such as heparan sulfate (HS), are significantly degraded, leading to impaired mechanosensory function (Davies et al., 2013; Jiang et al., 2022). More seriously, low-intensity and non-directional mechanical stimuli (Low FSS and OSS) induce phenotypic transformation in endothelial cells. This process manifests not only as widespread activation of the NF-κB signaling pathway (He et al., 2022), such as the release of pro-inflammatory factors induced by upregulation of the C-X3-C chemokine receptor 1 (Partridge et al., 2007; Yang et al., 2025); but also involves the abnormal mechanical microenvironment directly downregulating the synthesis of vasodilators such as eNOS and prostacyclin (PGI2), while significantly upregulating the expression of endothelin-1 (ET-1), vascular cell adhesion molecule-1 (VCAM-1), and platelet-derived growth factor (PDGF) (Malek et al., 1999). Furthermore, low shear stress increases the turnover rate and apoptotic tendency of endothelial cells, promoting a pathological state in the local microenvironment characterized by procoagulant and proliferative effects (Cho et al., 1997). This comprehensive functional shift of endothelial cells from “anti-inflammatory and vasodilatory” to “pro-inflammatory, vasoconstrictive, and proliferative”—triggered by mechanical signaling dysregulation (macroscopic flow field disturbance)—is a key physical factor underlying early endothelial dysfunction and focal progression of lesions in atherosclerotic ED (Wang et al., 2023).

### 4.2 Hypertension: endothelial mechanosensory desensitization under high stress loads

Endothelial cells play a central role in maintaining the tonicity of cavernous vessels and hemodynamic homeostasis by precisely sensing FSS and triggering NO-mediated flow-induced vasodilation; however, this physiological balance is significantly disrupted during hypertension-induced pathological remodeling (Félétou et al., 2010; Baeyens et al., 2016). Microvascular remodeling caused by long-term hypertension (such as luminal narrowing and an increased wall-to-lumen ratio) significantly distorts the stress distribution within the corpus cavernosum (Mulvany, 2002). At the pathomechanical level, high-pressure fluid not only increases the circumferential tension of the vascular wall but also leads to high-frequency, non-physiological pulsatile shear stress within the cavernous sinuses (Mulvany, 2002; Coste et al., 2010). This abnormal mechanical load significantly upregulates and chronically overactivates force-sensitive ion channels such as Piezo1 on the surface of endothelial cells, resulting in abnormal influx of extracellular Ca^2+^ (Wang et al., 2021). Although Piezo1-mediated Ca²^2^ flux is a necessary prerequisite for activating eNOS to release NO under physiological conditions, in a hypertensive environment, this sustained ionic overload triggers a severe oxidative stress cascade, inducing NADPH oxidase to produce excessive ROS (Porto Ribeiro et al., 2022; Backston et al., 2025). The accumulation of ROS leads to the depletion of BH4, a key cofactor of eNOS, which in turn triggers eNOS “decoupling”. As a result, endothelial cells not only lose their ability to produce NO but instead generate more superoxide anions, further impairing endothelial function (Förstermann et al., 2017). This eNOS-uncoupling/BH4-depletion axis is well established in systemic vascular biology; its direct demonstration in cavernosal endothelium is still lacking, and its occurrence in CCECs is therefore inferred rather than proven (Förstermann et al., 2017). Therefore, we hypothesize that the disruption of oxidative stress-mediated signaling pathways induces “mechanosensory desensitization” in the cavernous endothelium, compromising NO-dependent vasodilation in response to blood flow pulses during the early stages of erection, thereby constituting the physiological basis for the diminished response to normal hemodynamic stimuli in patients with hypertensive ED.

### 4.3 Diabetes: a “mechanical sensitivity blind spot” induced by high glucose levels

Diabetes causes systemic and multi-targeted damage to the vascular mechanical system (Martinez-Vidal et al., 2021). Hyperglycemia-derived advanced glycation end products (AGEs) degrade the endothelial glycocalyx and induce endothelial cell apoptosis (Liu et al., 2020), leading to shedding of the glycocalyx and “mechanical desensitization” of endothelial cells (Martinez-Vidal et al., 2021). Furthermore, AGEs promote pathological cross-linking of the extracellular matrix (ECM), increasing matrix stiffness and inhibiting the normal degradation of the ECM by matrix metalloproteinases (MMPs), thereby blocking fluid shear-mediated vascular biomechanical remodeling and dilation (Castela and Costa, 2016; Martinez-Vidal et al., 2021). Furthermore, PECAM-1 is an intercellular adhesion molecule enriched on the surface of CCECs, whose cytoplasmic domain contains phosphorylatable serine and tyrosine residues. Under fluid shear stress, tyrosine residues can be phosphorylated (Fleming et al., 2005; Tzima et al., 2005), which in turn activates downstream protein kinase B (Akt), promotes eNOS phosphorylation (Fleming et al., 2005), In addition, other adhesion proteins that interact with PECAM-1, such as VEGFR2 and VE-cadherin, are present at the adhesion junctions and jointly participate in the force transmission process of shear stress (Tzima et al., 2005; Coon et al., 2015). However, under diabetic pathological conditions, abnormally elevated oxidative stress (excessive ROS production) severely disrupts the downstream effects of this force-signal coupling pathway. Studies have shown that excessive ROS not only leads to “decoupling” of eNOS but also scavenges NO already generated by CCECs, thereby significantly impairing endothelium-mediated vasodilation (Wessells et al., 2006; Förstermann et al., 2017). The oxidative decoupling of eNOS is well documented in systemic endothelium; in CCECs it remains inferred, although hyperglycemia directly inhibits shear-induced NO production in human cavernosal endothelial cell (Wessells et al., 2006). Concurrently, abnormally elevated levels of thromboxane A2 (TxA2), derived from cyclooxygenase-2 (COX-2), further counteract the shear-induced vasodilatory effects (Bivalacqua et al., 2003; Castela and Costa, 2016). Thus, diabetes not only deprives CCECs of their mechanosensory structure—the glycocalyx—at the upstream level, disrupting PECAM-1 complex signaling, but also depletes NO via ROS and induces ECM stiffening through AGEs at the downstream level. This comprehensive dysfunction, ranging from mechanosensory perception to structural remodeling, constitutes a key pathophysiological basis for the loss of responsiveness to blood flow pulses in patients with diabetic ED.

### 4.4 Aging and microvascular stiffening: “functional blunting” of the endothelial mechanosensory system in the corpora cavernosa

Aging is an independent risk factor for ED (Zhuang et al., 2025). Unlike acute injury, the effects of aging on cavernous tissue manifest as a long-term biophysical remodeling. With advancing age, the proportion of smooth muscle in the corpus cavernosum decreases, while collagen fibers increase compensatorily and undergo abnormal cross-linking (e.g., mediated by AGEs), causing the physical properties of the corpus cavernosum microenvironment to shift from “high elasticity” to “high stiffness” (Pinheiro et al., 2000; Ferreira et al., 2014). This change in the “solid mechanics” environment interferes with the endothelial cells’ perception of FSS through mechanical transmission (Lai et al., 2026). First, aging significantly impairs the “front-end reception” of mechanical signals. The thickness of the EG and its core components (such as heparan sulfate) decline with age in multiple vascular beds (Sun et al., 2023); whether the cavernosal EG undergoes equivalent age-related thinning is reported only indirectly and warrants direct confirmation. This deterioration in mechanical sensing renders CCECs “insensitive” to blood flow shear stress during the initiation of erection, preventing them from efficiently capturing initial FSS signals (Kohn et al., 2015). Furthermore, aging-associated oxidative stress induces F-actin rearrangement in the endothelial cytoskeleton, increasing the cells’ intrinsic mechanical stiffness (Romagnoli et al., 2026). According to the theory of mechanical equilibrium—established in non-cavernosal endothelial models—increased endothelial cell stiffness generates higher “pre-stress”, which we hypothesize significantly raises the mechanical threshold required for mechanosensors (such as Piezo1 channels) to undergo conformational changes in CCECs (Kohn et al., 2015; Lai et al., 2023). Furthermore, aging disrupts the “intracellular transduction” of mechanical signals (Lai et al., 2026). In aged endothelial cells, the frequency of FSS-induced calcium influx decreases, and the coupling efficiency between Piezo1 channels and downstream eNOS is reduced (Lai et al., 2023); the same functional blunting in aged CCECs is hypothesized but not yet directly documented. Stiffness-dependent YAP/TAZ activation may likewise reinforce the pro-fibrotic loop in aged cavernosal tissue, although this extrapolation awaits direct testing (Wang et al., 2016; Wang et al., 2016). This “functional desensitization” of the mechanosensory system results in the inability of endothelial cells to produce sufficient NO to maintain an erection, even under adequate blood flow pressure (Vessières et al., 2012; Kohn et al., 2015). This vicious cycle, driven by matrix stiffening and mediated by the failure of endothelial mechanosensitivity, constitutes a key pathway in the transition of age-related ED from a biophysical to a biochemical process, and provides potential targets for clinical interventions targeting the mechanosensory pathway.

## 5 Conclusion and future directions

FSS is not only a passive physical signal that maintains vascular homeostasis in the corpus cavernosum of the penis, but also a key active regulator that drives CCECs to release NO and sustain erectile function. CCECs rely on a highly integrated mechanosensory system-including the apical glycocalyx-to efficiently convert dynamic hemodynamic signals into biochemical signals. This physiological process relies on the vascular endothelium maintaining a healthy “mechanically protected phenotype”, which involves the sustained release of vasodilatory and anti-inflammatory factors under stimulation by high-intensity laminar shear stress (>15 dyn/cm²) (Cheng et al., 2023). However, the occurrence of vascular-origin ED essentially reflects a systemic disruption in the coordination of this “force-chemical coupling” system, with its causes stemming from pathological remodeling of the biophysical microenvironment. Under pathological conditions, atherosclerosis-induced “local turbulent flow” drives the endothelium toward a pro-inflammatory and pro-coagulant phenotype (Cunningham and Gotlieb, 2005); the degradation of the glycocalyx caused by hyperglycemia, in turn, disrupts the mechanical sensing structures on the endothelial surface from the front end, rendering it unable to efficiently capture FSS signals (Liu et al., 2020). At a more fundamental biomechanical level, the increased matrix stiffness resulting from aging and fibrosis generates pathological “prestress”, which physically constrains the conformational switching of mechanoreceptors through skeletal rearrangement, leading to severe “mechanical perception blunting” in the corpus cavernosum in response to the initial blood flow pulse during erection (Martinez-Vidal et al., 2021). This progression from impaired physical signal transduction to the depletion of downstream biochemical output effects constitutes the biomechanical basis for the intractability of vasculogenic ED.

Although current studies have preliminarily elucidated the molecular network through which FSS regulates CCEC function, the existing literature still exhibits significant methodological limitations. Current conclusions regarding endothelial cell mechanosensing largely stem from traditional two-dimensional parallel-plate flow chamber or cone-plate viscometer models. Although these devices can provide precise steady-state laminar or pulsatile flow, they struggle to accurately replicate the highly complex hemodynamic microenvironment within the cavernous sinuses of the penis. First, the cavernous sinuses constitute a three-dimensional, porous, interwoven network, with CCECs growing on a collagen matrix possessing specific elasticity. In contrast, traditional two-dimensional rigid substrates (such as glass or plastic) significantly alter the physical constraints and “prestress” state of the cells at the substrate, which may lead to a shift in the thresholds of mechanoreceptors such as Piezo1. Second, the erectile process is accompanied by instantaneous blood flow pulses and extremely high intracavernosal pressure (ICP). This unique mechanical coupling pattern of “transient high shear stress combined with sustained high hydrostatic pressure” is difficult to simulate using existing conventional *in vitro* fluidic devices. Therefore, there is an urgent need to develop a “Cavernosum-on-a-chip” based on microfluidics technology. By integrating a three-dimensional matrix with adjustable stiffness, a co-culture system of primary CCECs and smooth muscle cells, and a pulsatile fluid-driven pump, this approach aims to precisely investigate the biomechanical pathogenesis of vascular-origin ED in an *in vitro* microenvironment that more closely mimics physiological conditions. Encouragingly, advanced biomimetic vascular flow models have already been used to dissect how hemodynamic profiles regulate endothelial inflammation in other vascular beds (He et al., 2024), supporting the feasibility of extending such platforms to the corpus cavernosum. Complementary technologies are now poised to characterize the genetic and physical changes induced by FSS in the corpus cavernosum. Single-cell RNA sequencing of human cavernosal tissue has already resolved endothelial, smooth-muscle, fibroblast and immune subpopulations and their altered intercellular communication in ED patients (Zhao et al., 2022), providing a transcriptional foundation onto which mechanosensitive gene signatures can be mapped. Atomic force microscopy (AFM) permits nanoscale measurement of endothelial cell stiffness and its remodeling by shear stress (Sato et al., 2000), quantifying the physical (not only transcriptional) fingerprint of disturbed flow. Fluorescence-based sensors—including FRET tension sensors engineered into PECAM-1 and VE-cadherin, which have visualized shear-induced changes in molecular tension across the junctional complex (Conway et al., 2013)—offer a route to watching mechanotransduction in living cells in real time; adapting these sensors to CCECs would directly test the hypotheses advanced in this review. The limits of current two-dimensional flow-chamber models in capturing endothelial senescence programs have been discussed elsewhere (Jimenez Trinidad et al., 2021). Looking ahead, the central challenge in the biomechanics of the corpus cavernosum lies in elucidating the “solid-fluid coupling” mechanism-specifically, how mechanical stresses in the solid matrix and shear forces from dynamic fluids jointly drive the phenotypic fate determination of endothelial cells through complex crosstalk between Piezo1, integrins, and the cytoskeleton. Future research should focus on the spatiotemporal regulatory logic of these mechanosensory molecules and explore how physical stimuli overcome mechanical barriers to translate into long-term biochemical remodeling of the tissue. At the clinical translation level, traditional hemodynamic indices (such as peak systolic velocity [PSV]) often lag substantive tissue lesions. Therefore, the development of non-invasive techniques, such as shear-wave elastography (SWE), to quantitatively assess the stiffness and viscoelastic parameters of the corpus cavernosum holds promise for a paradigm shift from mere “flow velocity monitoring” to “tissue biomechanical assessment”, providing a scientific basis for early and precise warning of vascular ED. Furthermore, intervention strategies targeting mechanical sensing failure are emerging as new breakthroughs: on the one hand, utilizing small-molecule agents to specifically repair damaged glycocalyx structures or restore the sensitivity of desensitized Piezo1 channels can reconstruct the endothelium’ s ability to respond to blood flow at the sensory level; on the other hand, “mechanotherapy”—centered on low-energy shockwave therapy (Li-ESWT)—holds promise for breaking through the physical constraints imposed by matrix stiffness through exogenous physical stress, thereby reshaping the damaged force-transduction network. Early clinical evidence supports this direction: in a pilot study of men with organic ED, Li-ESWT improved erectile function scores and penile endothelial function at 6-month follow-up (Vardi et al., 2010). Low-intensity pulsed ultrasound (LIPUS) similarly delivers exogenous mechanical stimuli that regulate cell fate through mechanotransduction—including Piezo1, integrin and calcium signaling—and is advancing rapidly in regenerative medicine (Lei et al., 2025), supporting the feasibility of ultrasound-based mechanotherapy for cavernosal vascular repair. Pharmacological repair of the glycocalyx is another emerging avenue—in patients with type 2 diabetes, short-term sulodexide partially restored glycocalyx dimensions and reduced vascular permeability (Broekhuizen et al., 2010)—and Piezo1 agonists such as Yoda1 relax cavernosal tissue ex vivo (Dela Justina et al., 2023), illustrating that the mechanosensory axis is druggable at multiple levels. In summary, reconstructing the diagnostic and therapeutic framework for ED from a biomechanical perspective not only deepens our understanding of the disease mechanism but also holds the potential to open new avenues for treatment, ranging from the repair of the physical microenvironment to functional regeneration.
